# The Hidden Burden of Water‐Binding Additives in Meat Products: Biochemical, Clinical, and Psychosocial Implications

**DOI:** 10.1002/mnfr.70591

**Published:** 2026-08-18

**Authors:** Oleksandr Kamyshnyi, Iryna Halabitska, Magnar Bjorås, Ingrid Bjørnes Sæther, Trygve Brautaset, Iryna Kamyshna, Oleh Lushchak, Valentyn Oksenych, Pavlo Petakh, Denis E. Kainov

**Affiliations:** ^1^ Department of Microbiology Virology and Immunology I. Horbachevsky Ternopil National Medical University Ternopil Ukraine; ^2^ Kyiv School of Economics Kyiv Ukraine; ^3^ Department of Therapy and Family Medicine I. Horbachevsky Ternopil National Medical University Ternopil Ukraine; ^4^ Department of Clinical and Molecular Medicine Norwegian University of Science and Technology Trondheim Norway; ^5^ Department of Microbiology Oslo University Hospital and University of Oslo Oslo Norway; ^6^ Centre of Healthy Embryology (CRESCO) Oslo Norway; ^7^ Department of Medical Rehabilitation I. Horbachevsky Ternopil National Medical University Ternopil Ukraine; ^8^ Department of Biochemistry and Pharmacology Uzhhorod National University Uzhhorod Ukraine

**Keywords:** carcinogenesis, food safety, food additives, metabolic syndrome, processed meat

## Abstract

Processed meat products are widely consumed worldwide, especially because they are affordable and easy to prepare. Their production often involves the use of water‐binding additives such as phosphates, nitrites, and sodium chloride. Although these substances improve taste, texture, and shelf life, their potential effects on human health remain unclear. This review aims to summarize current knowledge about the possible biological and clinical effects of these additives. Injection (brining) technology is commonly used to increase product weight and improve quality. However, it may also lead to regular consumption of these additives. Phosphates may influence mineral balance and could be linked to changes in vitamin D metabolism and vascular health. Nitrites can participate in the formation of N‐nitroso compounds, which have been shown to have harmful effects in experimental studies. High sodium intake is associated with increased blood pressure and cardiovascular risk. Some studies also suggest that these additives may affect the gut microbiota and promote low‐grade inflammation. Certain groups, including people with chronic diseases, may be more sensitive to these effects. Cultivated meat has been proposed as a potential alternative that may reduce reliance on such additives; however, it often still requires similar ingredients to achieve desired texture and stability, and its potential health benefits remain uncertain. Current regulations in the United States and the European Union primarily focus on individual additives and do not fully account for total daily intake from different foods. In addition, food labels often do not provide detailed quantitative information. Further research is needed to better understand long‐term effects and combined exposure.

## Graphical abstract

Graphical abstract illustrating how brining technology introduces water‐binding additives into processed meat. Their cumulative exposure may promote oxidative stress, inflammation, dysbiosis, and endothelial dysfunction, contributing to cardiovascular disease, chronic kidney disease, colorectal cancer, and metabolic burden, while highlighting the need for improved labeling and safer formulations.

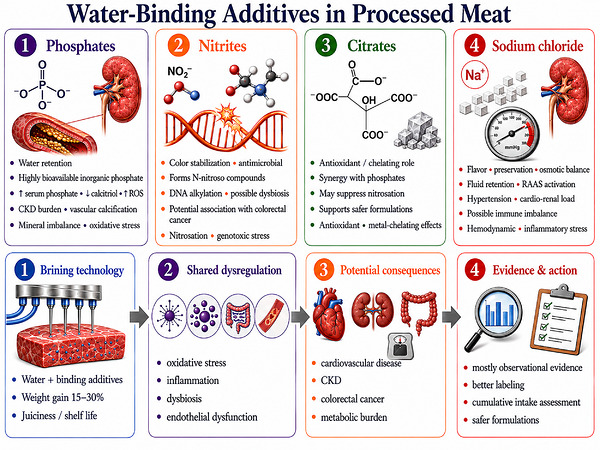

## 1 Food Additives in Processed Meat Products: Prevalence, Technological Applications and Health RSisks

Processed meat products are among the most widely consumed foods in the world, especially in cities and among lower‐income populations, where their relative accessibility and ease of preparation determine a sustainable demand [1, 2]. The meat processing industry actively uses moisture‐retaining food additives, in particular, phosphates, nitrites, and injectable salt solutions, which significantly improve the organoleptic properties and technological characteristics of the finished product [3, 4]. These substances increase the product's weight by approximately 15%–30%, enhance juiciness, and extend shelf life. At the same time, their use can reduce the food and nutritional value of the product by diluting the protein content and changing the structure of the tissues [5, 6, 7]. Despite its prevalence, this practice remains little known to the general consumer due to insufficient labeling and gaps in regulatory requirements [8].

Concerns about the effects of consuming such products gained clear scientific support in 2015, when the International Agency for Research on Cancer (IARC) of the World Health Organization classified processed meat as a Group 1 carcinogen, i.e., a substance with a proven link to the development of malignant neoplasms in humans. In particular, a statistically significant association was found between regular consumption of such products and an increased risk of colorectal cancer (CRC) [9]. In parallel, the European Food Safety Authority (EFSA) emphasized that dietary phosphate intake among the EU population often exceeds safe levels, particularly for vulnerable groups such as children, the elderly, and people with chronic kidney disease [10, 11, 12]. From a biochemical perspective, individual classes of food additives exert their negative effects through distinct molecular mechanisms [13, 14]. Excess dietary phosphate intake has been associated with disruptions in mineral homeostasis, including alterations in parathyroid hormone and fibroblast growth factor 23 (FGF‐23), and has been linked to vascular calcification and increased cardiovascular risk [15]. Nitrites, in turn, react with secondary and tertiary amines in the acidic environment of the stomach or during endogenous metabolism to form N‐nitroso compounds, a class of substances with established mutagenic and carcinogenic potential that can induce DNA alkylation in colonic mucosal cells [16, 17]. Finally, high sodium concentrations in processed meat products are associated with activation of the renin–angiotensin–aldosterone system, leading to increased blood pressure and fluid retention and placing a greater burden on the cardiovascular and renal systems [18, 19, 20].

Epidemiological evidence supports and extends these mechanistic observations. Large prospective cohort studies have consistently reported associations between regular consumption of processed meat and increased risk of obesity, cardiovascular disease, chronic kidney disease, and colorectal cancer; however, causal relationships remain uncertain [16, 21, 22, 23]. However, certain population, particularly those with pre‐existing renal dysfunction, metabolic syndrome, or genetic predisposition to dysregulation of calcium and phosphorus metabolism—are more susceptible to these effects, suggesting the need for differentiated health care approaches [21, 24, 25, 26]. Taken together, these data suggest that the impact of food additives in processed meat on human health is not only a toxicological but also a systemic health and social problem [27, 28]. Deepening knowledge about the molecular mechanisms of their action is a prerequisite for scientifically substantiating regulatory policy, improving labeling standards, and formulating evidence‐based dietary recommendations, especially for vulnerable populations [21, 29].

Unlike previous reviews that primarily focus on individual additives or specific disease outcomes, this review integrates the cumulative and potentially synergistic effects of multiple food additives commonly present in processed meat. In particular, it emphasizes the concept of cumulative exposure and the combined biological impact of phosphates, sodium, and nitrites across multiple organ systems.

A key contribution of this review is the conceptualization of processed meat additives not as isolated risk factors, but as components of a cumulative exposure system that may exert synergistic biological effects across metabolic, cardiovascular, and oncological pathways.

## 2 Methods

This study is a narrative review with elements of a structured literature search. Its aim is to summarize current evidence on the biochemical, clinical, and possible psychosocial effects of water‐binding additives in processed meat products, such as phosphates, nitrites, and sodium chloride.

A literature search was carried out using the databases PubMed/MEDLINE, Scopus, and Web of Science. We included studies published between January 2000 and January 2026. The search used the following keywords: “processed meat”, “food additives”, “phosphates”, “nitrites”, “sodium intake”, “gut microbiota”, “cardiovascular disease”, “chronic kidney disease”, “colorectal cancer”, “ultraprocessed food”, and “cultivated meat”. These terms were combined using the operators AND and OR.

We included peer‐reviewed articles published in English. The selected studies included human epidemiological studies, experimental studies (both in vivo and in vitro), and systematic reviews and meta‐analyses. We focused on studies that examined the biological, clinical, or microbiome‐related effects of food additives. We excluded nonpeer‐reviewed sources, articles not related to processed meat or additives, and studies with unclear methods.

This review has some limitations. As a narrative review, it may be subject to selection bias. In addition, most human data are observational, making it difficult to establish cause‐and‐effect relationships. Differences between studies also limit direct comparison of results.

## 3 Biochemical and Microbiological effects

### 3.1 Injection (Brining) Technology

**FIGURE 1:**
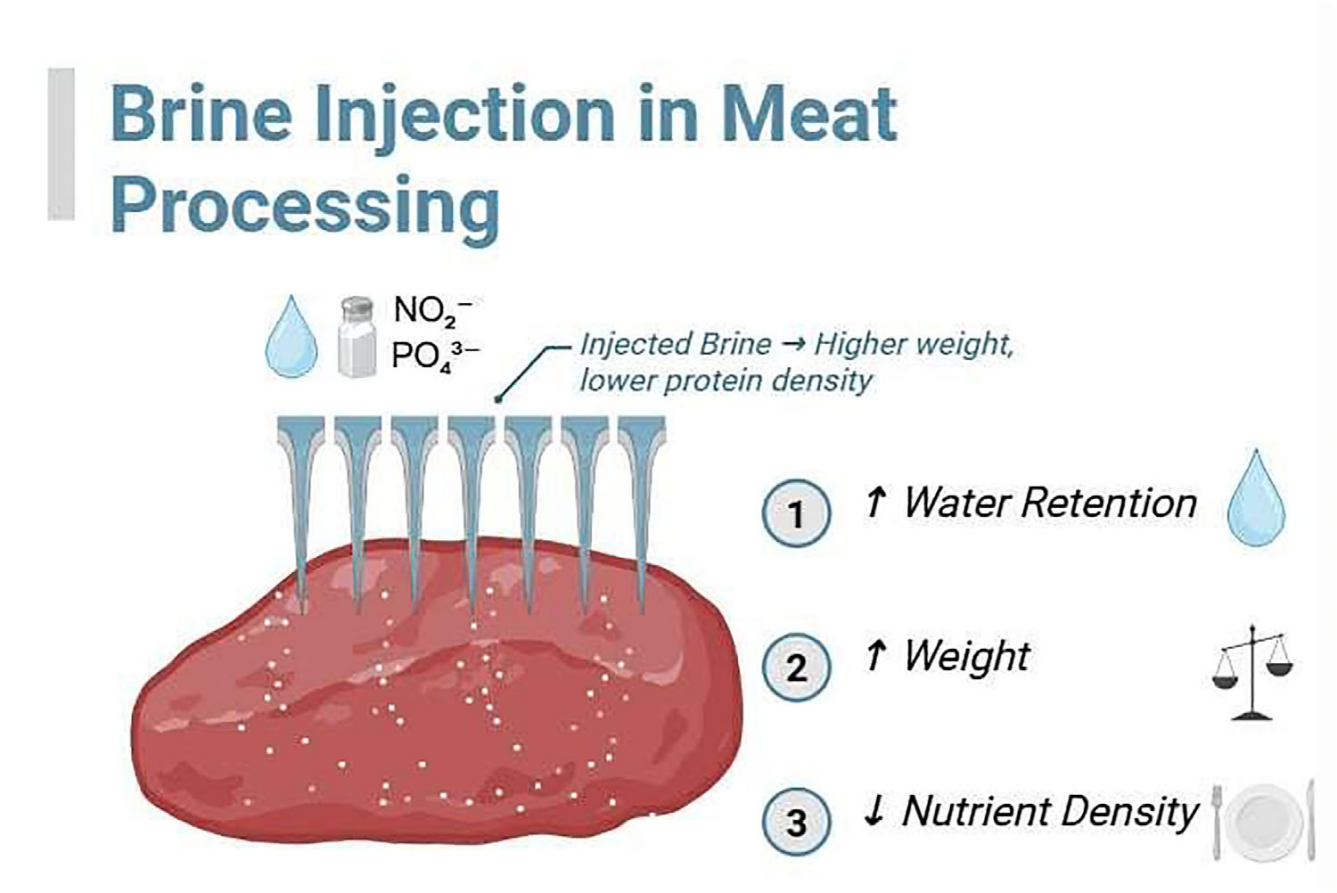
Brine Injection in Meat Processing Illustration of hollow needles injecting brine (water, sodium chloride, phosphates, nitrites) into muscle fibers, increasing water retention and product weight while diluting nutritional value. This figure highlights the industrial process driving additive exposure.

Injection is one of the most common meat processing technologies used to improve product quality and ensure its microbiological safety (Table 1). The method consists of injecting a solution containing water and functional additives, in particular, sodium chloride, phosphates, and nitrites, directly into muscle tissue using specialized injector needles (Figure 1) [5, 30]. Uniform distribution of brine throughout the product allows for a shorter processing cycle than traditional salting and ensures reproducibility of organoleptic characteristics in industrial production [31, 32]. Despite the obvious technological advantages, this process is biochemically complex and can have both positive and potentially negative effects on the nutritional value of the finished product [33, 34].

**TABLE 1:** Composition of typical brines used in meat and poultry processing.

Component	Function	Typical Concentration (%)
Water [57], [58]	Bulk filler, increases weight	5–20
Sodium chloride [59, 60]	Flavor, osmotic balance, preservation	1–3
Phosphates [61, 62, 63]	pH buffering, water retention	0.3–0.5
Nitrites (NaNO_2_) [50, 64]	Color stabilization, antimicrobial	50–200 ppm
Citrates [65]	Antioxidant, synergy with phosphates	0.1–0.3
Protein hydrolysates [66, 67]	Improve texture, bind water	0.5–1

Phosphates play a central role in the mechanism of injection action on meat tissue. Due to their alkalizing effect, they increase the pH of muscle tissue, altering the spatial configuration of myofibrillar proteins, actin, and myosin [35, 36, 37]. In particular, an increase in pH above the isoelectric point of myosin increases the electrostatic repulsion between protein molecules, which may contribute to loosening of the myofibrillar network and expansion of the space between muscle fibers [38, 39]. As a result, meat acquires the ability to retain a significantly larger volume of moisture—both before and after heat treatment—which ensures increased juiciness and tenderness of the finished product [40, 41]. At the same time, excessive addition of phosphates can disrupt the product's mineral balance and increase dietary phosphorus intake, posing a potential risk for people with impaired kidney function and a tendency toward vascular calcification [42]. Nitrites perform several interrelated key functions in the composition of brine. First, they stabilize the pink‐red color of meat through the formation of nitrosomyoglobin, a stable pigment resulting from the interaction of nitrite with myoglobin in muscle tissue [43, 44]. This characteristic plays a significant role in consumer perception of product quality, as it is traditionally associated with freshness and an attractive presentation [45]. Second, nitrites exhibit pronounced antimicrobial activity, inhibiting the growth of dangerous pathogenic microorganisms, primarily *Clostridium botulinum*, and slowing down the processes of microbial spoilage by inhibiting the enzymatic metabolism of the bacterial cell [46, 47]. However, nitrites can react with secondary amines present in meat proteins, forming N‐nitroso compounds — a chemical class of substances with proven mutagenic and carcinogenic potential [48, 49]. This process occurs both directly in the product during heat treatment (endogenous nitrosation) and in the consumer's gastrointestinal tract with the participation of nitrosating bacteria, which is especially relevant in the context of chronic consumption of such products [45, 46]. These circumstances emphasize the need for strict control of nitrite concentration in brine formulations and the search for safer functional alternatives. Importantly, the biological effects of nitrites are strongly influenced by other ingredients present in meat formulations. Ascorbic acid (vitamin C) and sodium erythorbate inhibit N‐nitrosamine formation by accelerating nitric oxide generation and reducing nitrosating intermediates, whereas citrate contributes antioxidant and metal‐chelating properties that may further suppress nitrosation reactions. Consequently, the potential health impact of nitrites depends not only on nitrite concentration but also on the overall formulation and processing conditions [44, 49, 50]. Thus, injection technology is an effective, technologically sound means of improving the quality and safety of meat products [44, 51]. At the same time, its implementation requires careful control of the dosage of functional ingredients and a deep understanding of their biochemical and microbiological action [52, 53]. In particular, it is necessary to take into account that the simultaneous presence of phosphates, nitrites, and sodium chloride in the brine composition may cause synergistic or antagonistic effects at the molecular level, which are currently insufficiently studied [54, 55, 56]. Further research should combine the analysis of molecular mechanisms of action of individual additives with a comprehensive assessment of their impact on long‐term human health, especially in vulnerable populations.

Recent technological advances aim to reduce additive requirements while maintaining product quality and microbiological safety. These include low‐temperature fermentation using protective starter cultures, antioxidant‐assisted curing systems, precision brining technologies, and natural antioxidant formulations, all of which have demonstrated potential to decrease nitrite and phosphate usage without compromising product quality [50, 51, 56, 66].

### 3.2 Biochemical Transformations

Inorganic phosphates, which enter the body as part of processed meat products, exhibit significantly higher bioavailability than organically bound phosphorus from plant sources: their intestinal absorption reaches 80%–100%, whereas for phytate phosphorus it does not exceed 40%–60% [34, 68]. Such efficient absorption may contribute to a rapid, pronounced increase in blood serum inorganic phosphate concentration, associating with a cascade of compensatory endocrine responses. In particular, hyperphosphatemia inhibits the activity of renal 1α‐hydroxylase, a key enzyme that catalyzes the conversion of 25‐hydroxycholecalciferol to calcitriol (1,25‐dihydroxycholecalciferol), the active form of vitamin D [68, 69]. Calcitriol deficiency, in turn, may reduce intestinal calcium absorption and enhance secondary hyperparathyroidism, and has been associated with adverse effects on bone mineral density and an increased risk of osteopenia [69, 70, 71].

In parallel, excess blood phosphate directly stimulates the proliferation and phenotypic transformation of vascular smooth muscle cells, which acquire osteoblast‐like properties and begin to synthesize an extracellular matrix prone to mineralization [72, 73, 74]. This process has been associated with increased arterial stiffness, impaired vascular elasticity, and elevated cardiovascular risk (Table 2) [72, 75].

**TABLE 2:** Biochemical and microbial effects of water‐binding additives.

Additive	Main target	Mechanism of action	Health effect
Phosphates [97, 98]	Mineral metabolism	↑ Serum phosphate, ↓ calcitriol, ↑ ROS	CKD, vascular calcification, aging
Nitrites [99, 100]	DNA integrity, microbiota	Nitrosamine formation, dysbiosis	Potential association with CRC (observational evidence), possible gut barrier alterations
Sodium [101, 102, 103]	Fluid balance, microbiota	Water retention, microbiota shifts	Hypertension, obesity, immune dysregulation
Injected water [104]	Protein density	Dilution of nutrient content	Reduced dietary quality, malnutrition

At the cellular level, excess phosphate disrupts mitochondrial function by altering the permeability of the inner mitochondrial membrane and destabilizing the electron transport chain, leading to increased electron leakage and excessive production of reactive oxygen species (ROS) [76, 77, 78]. These unstable molecule, including superoxide anion, hydrogen peroxide, and hydroxyl radicals, can oxidize cell membrane lipids, proteins, and DNA molecules [79, 80, 81]. The accumulation of such damage may contribute to accelerated cellular aging and has been hypothesized to increase susceptibility to chronic diseases, including cancer (Table 2) [82, 83, 84]. Oxidative stress also activates proinflammatory signaling pathways, including NF‐κB, further exacerbating the low‐grade systemic inflammation associated with metabolic syndrome and atherosclerosis [85, 86, 87].

Nitrites in brines exert their adverse effects primarily by endogenously forming N‐nitroso compounds. Under acidic conditions in the stomach or with the participation of nitrosating bacteria in the colon, nitrites react with secondary and tertiary amines of meat proteins to form nitrosamines and nitrosamides [28]. Experimental in vitro studies and animal models have been shown in experimental systems to alkylate DNA in colonic mucosal cells, inducing mutations in tumor suppressor genes and potentially contributing to malignant transformation, consistent with the mechanistic basis for the association between processed meat consumption and an increased risk of colorectal cancer established by IARC [88, 89]. Excess sodium, an integral component of most brines, has an additional negative effect by impairing renal regulation of water‐salt balance [90, 91, 92]. Chronically elevated sodium intake inhibits the activity of Na^+^/K^+^‐ATPase in renal tubular cells, which increases sodium and water reabsorption and activates the sympathetic nervous system and the renin–angiotensin–aldosterone axis [93, 94]. The combined effect of these mechanisms is associated with sustained increases in blood pressure and cardiac preload, which may contribute over time to left ventricular hypertrophy and an increased risk of heart failure (Table 2) [95, 96].

### 3.3 Microbiota and Immune Dysregulation

Nitrites may indirectly influence gut and cardiovascular health; however, their role in TMAO metabolism remains incompletely understood [95, 105]. TMAO has been associated with inflammation and increases the risk of cardiovascular disease [106, 107, 108]. Phosphates also negatively affect the body, in particular the composition of the gut microbiota. Evidence from mouse and rat studies suggests that high‐phosphate diets promote the growth of pathogenic bacteria while reducing overall gut microbial diversity [109, 110, 111]. Reduced microbial diversity has been associated with impaired resilience of host defense mechanisms and altered immune balance, primarily in experimental animal studies [112, 113]. Excess sodium also affects the immune system. Both human intervention studies and experimental animal models have demonstrated activation of Th17 cells in response to high sodium intake [114, 115]. Excessive activation of these cells may contribute to tissue damage and the development of autoimmune disorders, as demonstrated mainly in experimental models [116, 117, 118, 119]. When these changes occur simultaneously, an imbalance in the gut microbiota results. The production of short‐chain fatty acids, such as butyrate, which normally support the strength and health of the intestinal mucosa, is reduced [120, 121, 122]. The lack of sufficient amounts of these acids weakens the intestinal barrier, making it more permeable to pathogens [123, 124, 125]. A weakened intestinal barrier may facilitate the translocation of microorganisms, which may be associated with increased susceptibility to infections [126, 127]. This may be associated with altered host defense mechanisms and potential changes in susceptibility to certain infections; however, direct clinical evidence remains limited [128, 129, 130, 131]. However, current evidence linking food additive exposure to microbiota‐mediated disease outcomes remains limited, and causal relationships have not been established in humans. Most available data are derived from experimental or animal studies, and their relevance to human health requires further investigation. Most evidence linking high sodium intake to gut microbiota alterations is derived from experimental animal studies, whereas direct evidence in humans remains limited. Nevertheless, excessive sodium intake is well established as an independent risk factor for hypertension, cardiovascular disease, and chronic kidney disease, providing a strong clinical rationale for limiting dietary sodium intake irrespective of its microbiota‐related effects.

## 4 Health and Psychosocial Impacts

### 4.1 Cancer Risk

The consumption of processed meat is one of the best‐documented dietary risk factors for colorectal cancer (CRC). Large human epidemiological studies and meta‐analyses suggest that this dietary pattern is responsible for approximately 10%–15% of CRC cases in developed countries [132, 133, 134]. The mechanistic basis for this association is the endogenous formation of N‐nitroso compounds in the acidic environment of the stomach and by nitrosating bacteria in the distal intestine [134]. Experimental animal and in vitro studies have shown that these compounds are associated with DNA alkylation in colonic mucosal cells, causing mutations in tumor suppressor genes, including APC, TP53, and MLH1, and thereby potentially contributing to malignant epithelial transformation [132, 135]. Regular and prolonged intake of nitrites, nitrates, and preservatives in processed meat creates a chronically unfavorable intestinal microenvironment that supports the proliferation of transformed cells and inhibits apoptosis [136, 137]. It remains challenging to distinguish the effects of specific additives from those of the processed meat matrix as a whole.

It is important to distinguish hazard identification from individual risk assessment. The IARC Group 1 classification reflects the strength of evidence that processed meat can cause cancer under conditions of sufficient exposure but does not imply that occasional consumption represents a high individual risk. Epidemiological studies indicate that elevated colorectal cancer risk is primarily associated with regular long‐term intake [22, 138].

### 4.2 Cardiovascular and Renal Consequences

**FIGURE 2:**
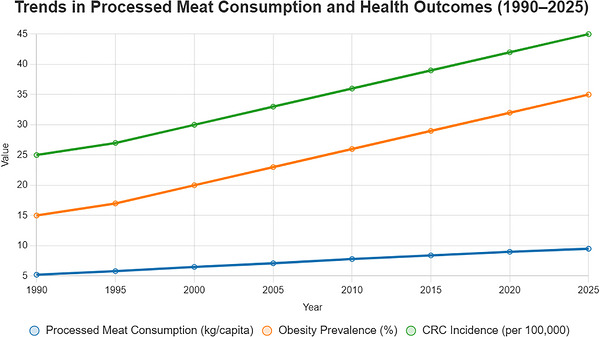
Trends in Processed Meat Consumption and Health Outcomes A line chart illustrating global processed meat consumption (kg per capita, 1990–2025) alongside rising obesity and CRC rates in high‐income countries. Data sources: FAO, WHO, GLOBOCAN. This figure highlights the parallel increase in additive exposure and disease burden.

Excessive phosphate intake is particularly dangerous for individuals with chronic kidney disease (CKD). Clinical studies in patients with CKD indicate that reduced renal phosphorus excretion contributes to persistent hyperphosphatemia, which has been associated with accelerated CKD progression by activating profibrotic signaling pathways, stimulating FGF‐23 synthesis, and creating a vicious cycle of mineral and bone disorders (Figure 2) [139, 140, 141]. In parallel, hyperphosphatemia enhances systemic inflammation by activating NF‐κB and increasing levels of proinflammatory cytokines‐particularly IL‐6 and TNF‐α. These findings have been reported in patients with CKD as well as supported by experimental studies, and further contribute to the progression of atherosclerosis and increased cardiovascular mortality in this patient group [139, 141, 142].

Excess dietary sodium and nitrites act synergistically in shaping cardiovascular risk. Chronically elevated sodium intake activates the renin–angiotensin–aldosterone system, increasing blood pressure and myocardial preload [88, 143]. Nitrites, in turn, have been shown in experimental animal and cellular studies to stimulate the production of proinflammatory eicosanoids and increase the expression of adhesion molecules on endothelial cells, facilitating the transmigration of monocytes into the vascular wall and accelerating the formation of atherosclerotic plaques [144, 145]. The combined effect of these mechanisms is associated with an increased risk of acute cardiovascular events—myocardial infarction and ischemic stroke—and also contributes to the development of endothelial dysfunction, which is an early and sensitive marker of vascular damage [88, 146, 147]. These associations may also reflect broader dietary patterns characterized by high intake of ultraprocessed foods.

### 4.3 Integrative Risk Assessment

**FIGURE 3:**
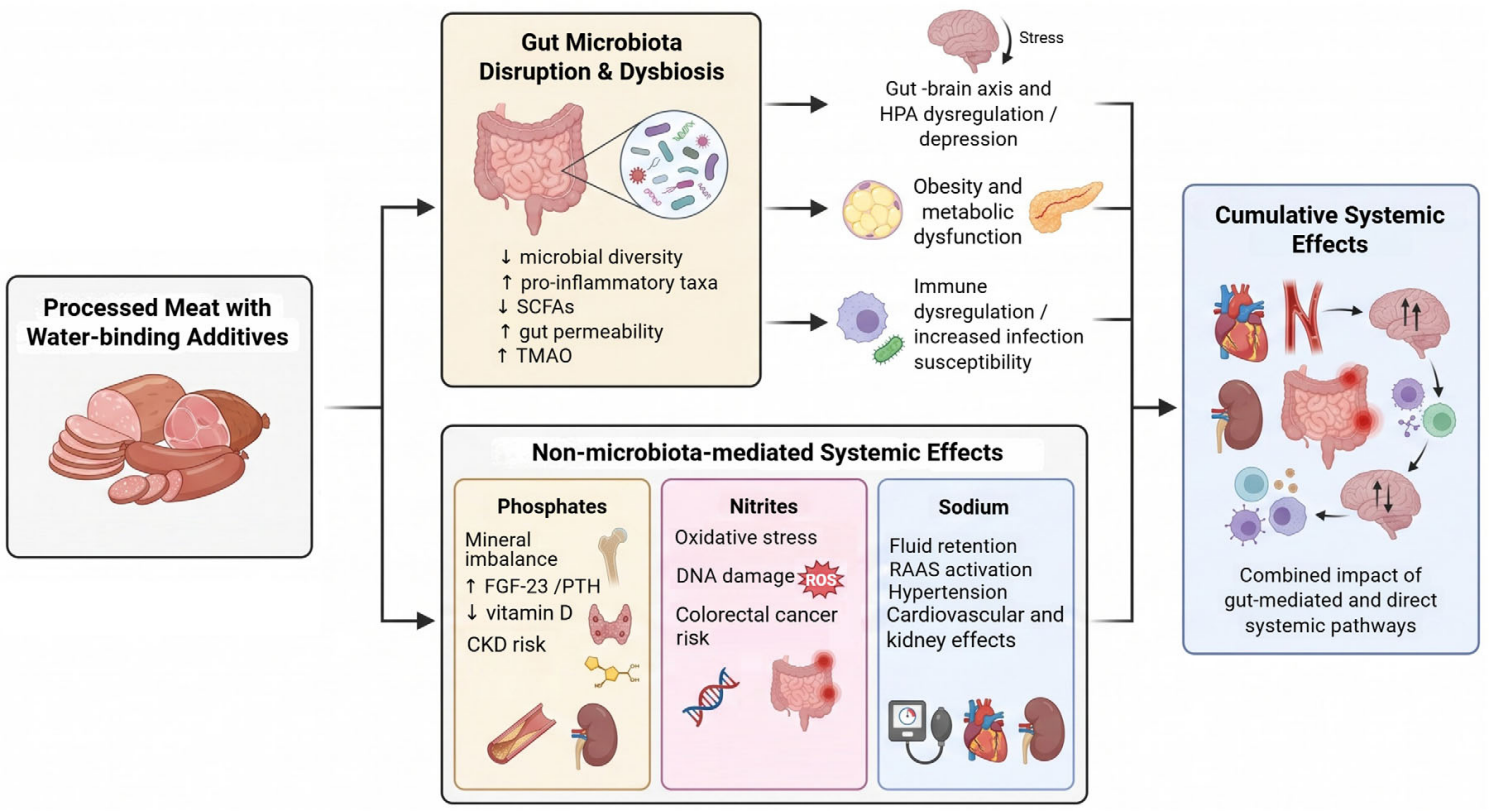
Pathways from Additives to Dysbiosis and Disease Flowchart depicting how water‐binding additives disrupt gut microbiota, leading to immune dysregulation, infection susceptibility, obesity (via inflammation, water retention), and depression (via gut–brain axis, HPA dysregulation).

The evidence presented demonstrates that excessive consumption of processed meat and its key components—phosphates, nitrites, and sodium—suggests a complex and potentially interconnected pathophysiological network that encompasses oncological, cardiovascular, metabolic, neuropsychiatric, and microbiome disruptions (Figure 3). It is important to emphasize that these effects are not independent: systemic inflammation, endothelial dysfunction, insulin resistance, and dysbiosis form mutually reinforcing feedback loops that significantly increase the overall burden of disease at the population level. The available evidence from human observational studies, supported by experimental animal and mechanistic investigations, supports consideration of limiting consumption of such products, particularly in high‐risk populations [128, 132, 139, 148, 149, 150].

Emerging evidence indicates that dietary interventions may attenuate some adverse biological effects associated with processed meat additives. Dietary fiber promotes short‐chain fatty acid production and supports intestinal barrier integrity, polyphenols reduce oxidative stress and inflammation, probiotics help restore gut microbial balance, whereas adequate vitamin D status may improve phosphate homeostasis. Although these interventions cannot eliminate additive exposure, they may reduce susceptibility to some downstream metabolic disturbances [151, 152, 153].

## 5 Cultivated Meat as a Potential Strategy to Reduce Additive‐Related Health Risks

Given growing concerns about the cumulative health effects of water‐binding additives in processed meat, there is increasing interest in alternative protein sources that may reduce reliance on such additives [154]. Cultivated meat has emerged as a promising approach that allows for controlled production conditions and the potential to minimize or optimize the use of functional additives [155, 156]. Therefore, examining the role of water‐binding additives in cultivated meat systems provides an important perspective on how future food technologies may address current health risks.

Cultivated meat is produced by growing cells in bioreactors as a more sustainable alternative to traditional meat [157]. It often has a lower water‐holding capacity (WHC) due to the immature tissue structure and high moisture content. This may lead to water loss during cooking, freezing, or reheating, thereby degrading dish quality. Water‐binding additives (WBA) help improve the juiciness, texture, and stability of the product [158].

The main WBAs include: Hydrocolloids (alginate, carrageenan, gellan gum, pectin, xanthan, and konjac‐glucomannan), Protein additives (transglutaminase (MTG), whey proteins, gelatin, and zein), and others (dietary fibers, modified starches, and crosslinking agents). They form gel structures, bind water, and mimic the natural extracellular matrix, stabilizing the product [159, 160].

Although hydrocolloids are frequently marketed as clean‐label alternatives, they cannot be regarded as biologically inert. Experimental studies suggest that carrageenan may impair intestinal barrier integrity and promote intestinal inflammation under certain conditions, whereas xanthan gum may cause gastrointestinal discomfort at high intake levels. Alginate is generally considered well tolerated, although it may influence gastric emptying and intestinal viscosity. Current human evidence remains limited, and further studies are required to clarify long‐term gastrointestinal effects [161, 162, 163].

WBAs increase water‐holding capacity and reduce cooking losses, maintaining juiciness. Alginate hydrogels improve structure, strength, and moisture retention. When combined with fats (alginate, MTG), they form a texture similar to natural meat, preventing delamination during heating. Hydrocolloids also contribute to the formation of a fibrous structure and increase tenderness. Dietary fibers and konjac glucomannan additionally retain water and improve product stability [164, 165, 166].

**FIGURE 4:**
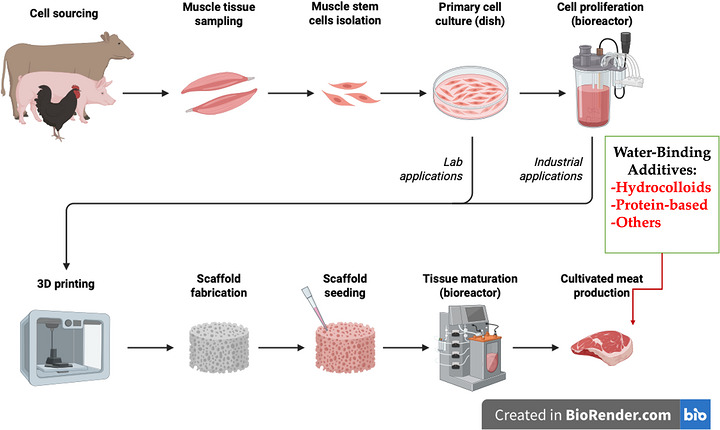
Integration of water‐binding additives into the cultured meat production process. The diagram illustrates the complete technological chain of cultured meat production—from cell selection and proliferation to tissue formation and obtaining the final product. Special attention is paid to the role of water‐binding additives, which are incorporated at various stages to enhance structure, stability, and water‐holding capacity. The combination of bioengineering approaches, 3D printing, and functional ingredients ensures a product with properties similar to traditional meat.

Excess hydrocolloids can make the texture too soft or gel‐like. The demand for a “clean label” limits the use of synthetic additives. In liquid dishes, stratification can occur if an excessive amount is added. The optimal concentration is usually 0.5%–3% (Figure 4) [167, 168, 169].

Recent research is aimed at creating natural and functional scaffolds (e.g., alginate and protein‐based) and hybrid products. This allows for cultured meat with better texture and juiciness for different dishes and meets sustainable and consumer requirements [164, 170, 171].

However, it should be emphasized that cultivated meat does not inherently eliminate the need for water‐binding additives. In many cases, hydrocolloids and protein‐based binders are still required to achieve desirable texture and stability. Therefore, the potential health benefits of cultivated meat depend not only on the production system itself but also on the formulation and regulatory oversight of additive use. Further research is needed to evaluate whether these systems truly reduce cumulative additive exposure compared to conventional processed meat. Moreover, the health implications of the combinations of additives used in cultivated meat products remain largely unknown and require further investigation. In addition, although production costs have decreased substantially in recent years, cultivated meat remains considerably more expensive than conventional meat, limiting its widespread commercial availability.

## 6 Regulatory Framework and Evidence Gaps

### 6.1 Current Regulatory Approaches

**FIGURE 5:**
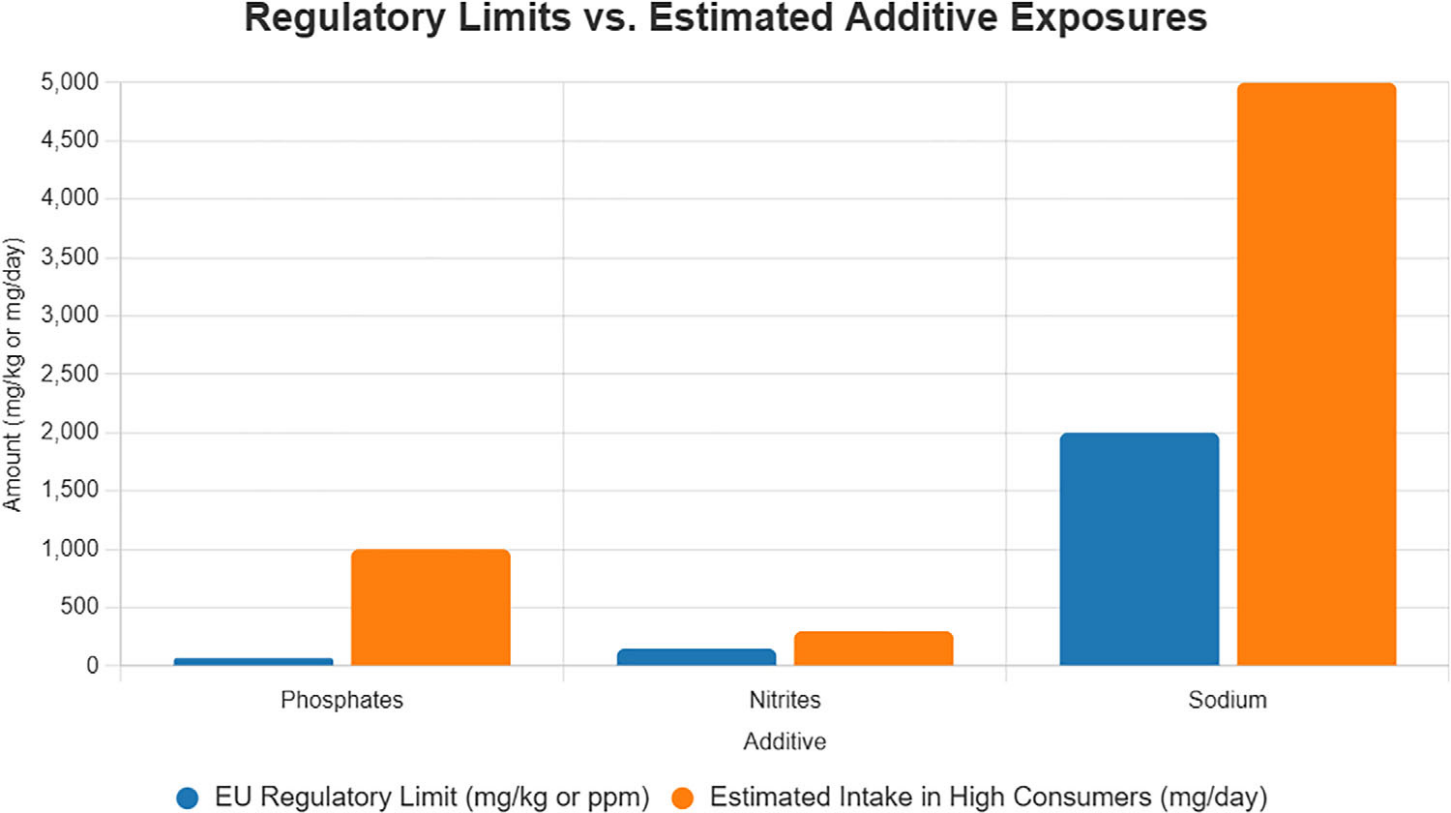
Regulatory Limits vs. Estimated Additive Exposures A bar chart comparing EU/US regulatory limits for phosphates, nitrites, and sodium with estimated intakes in high consumers, highlighting exposure excesses and the need for RCTs.

In the United States, the Food and Drug Administration (FDA) and the Food Safety and Inspection Service (FSIS) of the Department of Agriculture (USDA) jointly regulate food additives in meat products [172, 173]. Under current law, manufacturers are permitted to use a wide range of permitted additives, including phosphates, nitrites, and sodium chloride, in poultry and pork meat products without being required to fully disclose their quantitative composition on the label [174]. Labeling requirements are limited to listing ingredients in descending order of weight, which does not provide consumers with accurate information about the concentration of specific substances in the product. As a result, the vast majority of consumers are unable to assess their dietary exposure to these compounds (Figure 5) [174, 175].

The European Union regulatory system is more detailed: Regulation (EC) No 1333/2008 sets maximum permissible levels (MRLs) for phosphates and nitrites in different categories of meat products, and Regulation (EC) No 1169/2011 regulates consumer information [176, 177]. Despite this, the current regulatory framework has several limitations: the MRLs set relate to individual products and do not account for the total daily intake of these compounds from all food sources simultaneously. Since phosphates and nitrites are widely present not only in processed meat but also in soft drinks, dairy products, bakery products, and food supplements, the actual dietary exposure of the consumer may significantly exceed safe levels, even if each individual product formally complies with the established standards (Figure 5) [178]. This fragmentation of regulation effectively makes it challenging to accurately assess the overall risk for the population.

### 6.2 Evidence Gaps and Methodological Limitations

Limitations in the current evidence base and accumulated epidemiological data: none of the existing regulatory systems provides for the assessment of the potential effects of food additives on the immune system or mental health [179, 180, 181]. Meanwhile, some studies indicate a possible role for excess phosphate in modulating the immune response by altering dendritic cell function, and excess sodium is associated with the activation of Th17 lymphocytes and increased autoimmune responses. These areas remain outside the scope of current regulation due to insufficient evidence. Most of these findings originate from animal experiments and mechanistic in vitro studies, whereas confirmation in large human studies remains limited [182, 183, 184].

A fundamental methodological problem is the critical shortage of randomized controlled trials (RCTs) on the long‐term effects of food additives [185, 186]. Conducting such studies in food nutrition is associated with well‐known ethical and practical difficulties: the impossibility of controlling diet over a long period, low participant compliance, and significant variability in individual metabolism [182, 187]. The available evidence base is mainly based on cohort and case‐control studies, which allow to detect statistical associations between the level of consumption of certain substances and the risk of adverse health outcomes, but cannot establish a cause‐and‐effect relationship [188, 189]. Significant limitations of observational studies are residual confounding, errors in dietary history, and the inability to isolate the effect of a single supplement from the overall dietary pattern (Table 3).

**TABLE 3:** Current regulatory limits versus estimated exposures.

Additive	EU regulatory limit	US regulatory allowance	Estimated intake in high consumers
Phosphates [173, 176, 177]	40–70 mg/kg bw/day (ADI)	Allowed in brines (≤0.5%)	>1000 mg/day
Nitrites [173, 176, 177]	150 mg/kg in meat	Up to 200 ppm	>2x ADI
Sodium [173, 176, 177]	<2 g/day recommended	No strict cap	3–5 g/day
Water [173, 176, 177]	No limit	Permitted if labeled	Adds 15%–30% weight

Also worthy of special attention is the lack of standardized biomarkers to assess the chronic effects of phosphates and nitrites on the human body under real‐life nutritional conditions. Without validated exposure indicators, it is impossible to correctly verify doses in clinical trials and to set thresholds for regulatory purposes.

Most available data are derived from observational studies, which are subject to residual confounding, measurement error in dietary assessment, and limited ability to isolate the effects of individual additives

### 6.3 Prospects for Improving the Regulatory Framework

The identified gaps highlight the need for a fundamental rethinking of approaches to regulating food additives in meat products. First, regulators need to move from assessing the safety of individual products to a cumulative analysis of the total daily and long‐term exposure from all food sources of a substance, an approach already partially implemented by EFSA but requiring further operationalization. Second, labeling requirements need to be significantly strengthened: consumers should have access to quantitative information on the content of key additives, not just their presence in the product. Third, overcoming methodological limitations requires developing new study designs that combine Mendelian randomization, systems biology, and gut microbiome analysis to establish causal relationships between dietary exposures and clinical outcomes. Only a comprehensive scientific approach that integrates epidemiological observations, controlled experimental models, and multisystem assessment of effects—including metabolic, immune, and neurological—can provide the basis for developing evidence‐based regulatory standards and effective consumer education strategies.

It should be noted that regulatory limits for food additives are established based on toxicological safety assessments. Moderate consumption of processed meat within recommended dietary patterns is generally considered acceptable for the general population.

## 7 Conclusions

Available evidence suggests that water‐binding additives used in processed meat products may have an impact on human health through several biological pathways. These include possible effects on mineral metabolism, oxidative stress, blood pressure regulation, and gut microbiota composition. However, most current data come from observational and experimental studies, and clear cause‐and‐effect relationships have not been fully established.

The combined intake of phosphates, nitrites, and sodium from different food sources may be important, as their effects can interact. At the same time, individual risk may vary depending on overall diet, lifestyle, and pre‐existing health conditions. Certain groups, such as people with cardiovascular or kidney diseases, may be more sensitive to these exposures.

Cultivated meat has been proposed as a potential alternative that may reduce some risks associated with traditional processed meat. It allows for more controlled production conditions and may offer opportunities to optimize or limit the use of certain additives. However, it is important to note that water‐binding agents are often still required in these products to achieve acceptable texture and stability. Therefore, the potential health benefits of cultivated meat remain uncertain and depend on formulation and regulation.

Current regulatory systems mainly evaluate additives separately and at the level of individual products. This approach may not fully reflect real‐life dietary patterns, where multiple sources contribute to total intake. In addition, limited quantitative information on food labels can make it difficult for consumers to assess their exposure.

Future research should focus on the long‐term and combined effects of these additives, using more advanced and integrated approaches. Improving transparency in labeling and considering cumulative intake could support better risk assessment and more informed dietary choices.

## Funding

This work was supported by seed funding from the Norwegian University of Science and Technology (NTNU).

## Data Availability

The authors have nothing to report
